# A Color Image Encryption Algorithm Based on Double Fractional Order Chaotic Neural Network and Convolution Operation

**DOI:** 10.3390/e24070933

**Published:** 2022-07-05

**Authors:** Nanming Li, Shucui Xie, Jianzhong Zhang

**Affiliations:** 1 School of Communication and Information Engineering, Xi’an University of Posts and Telecommunications, Xi’an 710121, China; lnmwechat@163.com; 2 School of Science, Xi’an University of Posts and Telecommunications, Xi’an 710121, China; 3 School of Mathematics and Statistics, Shaanxi Normal University, Xi’an 710119, China; jzzhang@snnu.edu.cn

**Keywords:** fractional order chaotic system, neural network, convolution operation, DNA encoding, image encryption

## Abstract

A color image encryption algorithm based on double fractional order chaotic neural network (CNN), interlaced dynamic deoxyribonucleic acid (DNA) encoding and decoding, zigzag confusion, bidirectional bit-level diffusion and convolution operation is proposed. Firstly, two fractional order chaotic neural networks (CNNs) are proposed to explore the application of fractional order CNN in image encryption. Meanwhile, spectral entropy (SE) algorithm shows that the sequence generated by the proposed fractional order CNNs has better randomness. Secondly, a DNA encoding and decoding encryption scheme with evolutionary characteristics is adopted. In addition, convolution operation is utilized to improve the key sensitivity. Finally, simulation results and security analysis illustrate that the proposed algorithm has high security performance and can withstand classical cryptanalysis attacks.

## 1. Introduction

The rapid development of information technology has greatly facilitated people’s daily life, but the information security issues caused by this method cannot be ignored. Digital image, which is one of the information carriers, is extensively used in medical, education, military and other fields. However, the security of image information is hard to guarantee due to the openness of the internet platform. If the problem of image information security cannot be solved, it will cause irreparable loss to military, education, medical, and other fields. Therefore, it is of great significance to design a safe and effective image encryption algorithm. A puzzling phenomenon is that image information has the characteristics of high data redundancy, strong pixel correlation, and large data capacity, which makes the traditional encryption algorithms unsuitable for image encryption [1,2]. In view of the above information characteristics of image, image encryption algorithms based on different technologies have been extensively researched, including chaos theory [3,4,5,6], deoxyribonucleic acid (DNA) encoding and calculation [7,8], cellular automata [9,10], etc.

Chaotic system applies to image encryption because of its similar characteristics with cryptography, such as ergodicity, initial value sensitivity, and aperiodicity [11,12]. Common chaotic systems include Logistic mapping, Henon mapping, Lorenz chaotic system, Hopfield neural network (HNN) chaotic system, etc. Among them, the HNN model was proposed by the American physicist Hopfield in 1982 [13]. This model can generate very complex behaviors, such as hyper-chaos, and chaos, etc. Moreover, due to the nonlinear activation function of neurons, this model has a strong nonlinear characteristic. Therefore, HNN has been extensively researched and applied in image encryption [14,15,16]. In addition, fractional calculus has more than 300 years of theoretical research history, but it was not applied in engineering, physics or applied mathematics until recent decades [17]. Some studies show that, when introducing a fractional differential operator into chaotic system, the system will produce more accurate and complex dynamic behavior, and have stronger randomness and unpredictability [17]. Meanwhile, in contrast to an integer order chaotic system, the order of a fractional order chaotic system can also be used as the key of the encryption algorithm.

DNA operation is extensively used in image encryption because of its high parallelism [18], which can improve the efficiency of encryption and decryption. However, DNA operation is, in essence, a von Neumann operation principle [19]; its operation rules are well-known. Chen et al. [20] investigated the properties of DNA encoding and found that some encryption schemes based on DNA encoding have different degrees of insecurity. The image encryption algorithms proposed in [21,22] adopt fixed DNA encoding rules, that is, the encoding rules are not related to plain image, which will reduce the security of the encryption algorithm. Therefore, we need to do something else to make DNA operation more unpredictable to ensure the security of encryption algorithm. In addition, bit-level diffusion can better hide the information from each bit plane of image than pixel-level diffusion. Lately, some bit-level image encryption algorithms were proposed [21,23,24]. In [21], a color image encryption algorithm based on DNA operation and chaos was proposed, which adopts the fixed DNA encoding rule. In [23], a color image encryption algorithm based on DNA encoding and double chaos system was proposed, which adopts dynamic DNA encoding. In [24], two unidirectional diffusion algorithms are mentioned. However, the unidirectional diffusion algorithm cannot propagate the subtle change of plain image to every pixel of encrypted image by a round diffusion operation.

On the basis of the previous analysis, we propose a color image encryption algorithm based on the double fractional order chaotic neural network (CNN), interlaced dynamic DNA encoding and decoding, zigzag confusion, bidirectional bit-level diffusion, and convolution operation. This paper contributes the following aspects:

(1) Two fractional order CNNs are proposed. The chaotic performance analysis shows that a fractional order CNN has more complex chaotic behavior in comparison to the integer order CNN.
(2) A new bidirectional bit-level diffusion algorithm is applied. The new bidirectional bit-level diffusion algorithm can hide the bit-plane information of plain image better.
(3) An interlaced dynamic DNA encoding and decoding encryption scheme is adopted. This scheme has the evolution characteristic, which makes the encryption algorithm have higher security.
(4) In the permutation algorithm, a convolution operation is used to associate the permutation process with plaintext information, which greatly enhances the key sensitivity and plaintext sensitivity of the algorithm.

The rest of the paper is organized as follows. In Section 2, the CNN is introduced and its dynamic characteristics are analyzed. In Section 3, some fundamental knowledge is given. Section 4 describes the proposed algorithm. Section 5 presents the simulation results. Security analyses are placed in Section 6. The conclusion is provided in Section 7.

## 2. CNN

### 2.1. Integer Order CNN

Based on the HNN model, [25] proposed a chaotic neuron model, whose definition is

(1) $$\begin{array}{cc} c_{i}{\dot{x}}_{i}=\sum_{j=1}^{n}s_{ij}x_{j}+\sum_{j=1}^{n}w_{ij}v_{j}+d_{i}, & i=1,\cdots ,n. \end{array}$$

**Figure 1:**
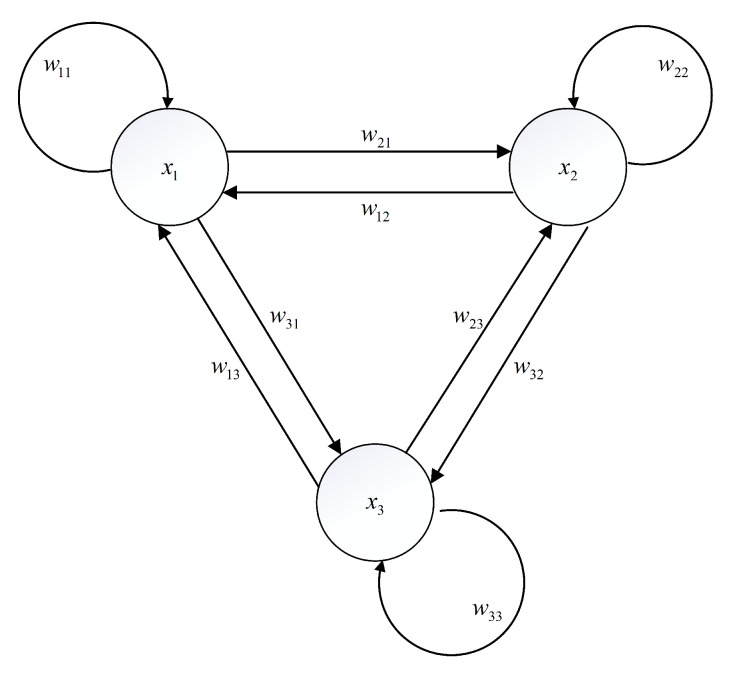
The connections between neurons in (2).

In this paper, we let $c_{i}$ = 1 and *n* = 3, so the integral order CNN is

(2) $$\begin{array}{cc} {\dot{x}}_{i}=\sum_{j=1}^{3}s_{ij}x_{j}+\sum_{j=1}^{3}w_{ij}v_{j}+d_{i}, & i=1,2,3. \end{array}$$

where $v_{j}=\tanh(x_{j})$. Figure 1 shows the connections between neurons in Equation (2). The connection weights $w_{ij}$, the conductance of membrane resistance $s_{ij}$ and input current $d_{i}$ in Equation (2) are determined as

(3) $$\begin{array}{ccc} s_{ij}=\left[\begin{array}{ccc} 0 & 2 & 0\\ 0 & 0 & 1\\ 0 & -3 & -5 \end{array}\right]; & w_{ij}=\left[\begin{array}{ccc} 2 & 1 & -9\\ -9 & 2 & 4\\ 1 & -9 & 2 \end{array}\right]; & d_{i}=\left[\begin{array}{c} 0\\ c\sin(x_{1})\\ 0 \end{array}\right]. \end{array}$$

So Equation (2) can be defined as

(4) $$\begin{array}{c} \left\{\begin{array}{c} {\dot{x}}_{1}=2x_{2}+2\tanh\left(x_{1}\right)+\tanh\left(x_{2}\right)-9\tanh\left(x_{3}\right);\\ {\dot{x}}_{2}=x_{3}-9\tanh\left(x_{1}\right)+2\tanh\left(x_{2}\right)+4\tanh\left(x_{3}\right)+c\sin\left(x_{1}\right);\\ {\dot{x}}_{3}=-3x_{2}-5x_{3}+\tanh\left(x_{1}\right)-9\tanh\left(x_{2}\right)+2\tanh\left(x_{3}\right). \end{array}\right. \end{array}$$

**Figure 2:**
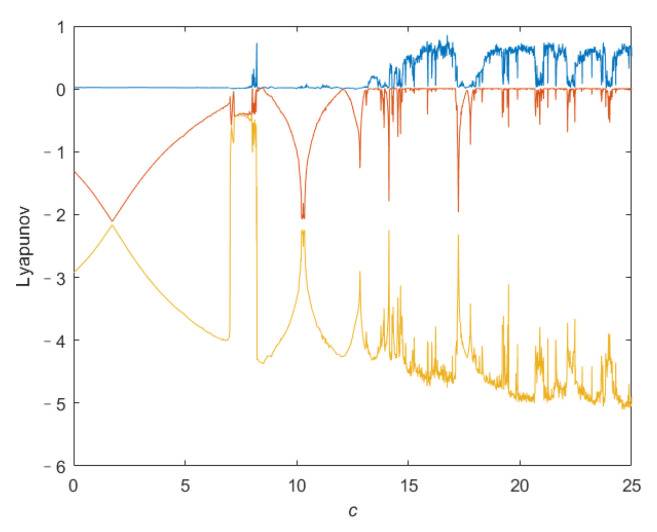
LE spectrum of the system (4).

**Figure 3:**
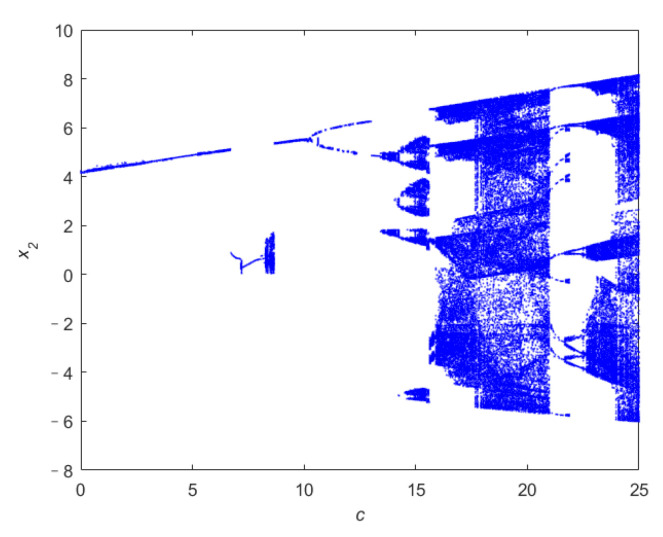
The $x_{2}$-axis bifurcation diagram of *c*.

**Figure 4:**
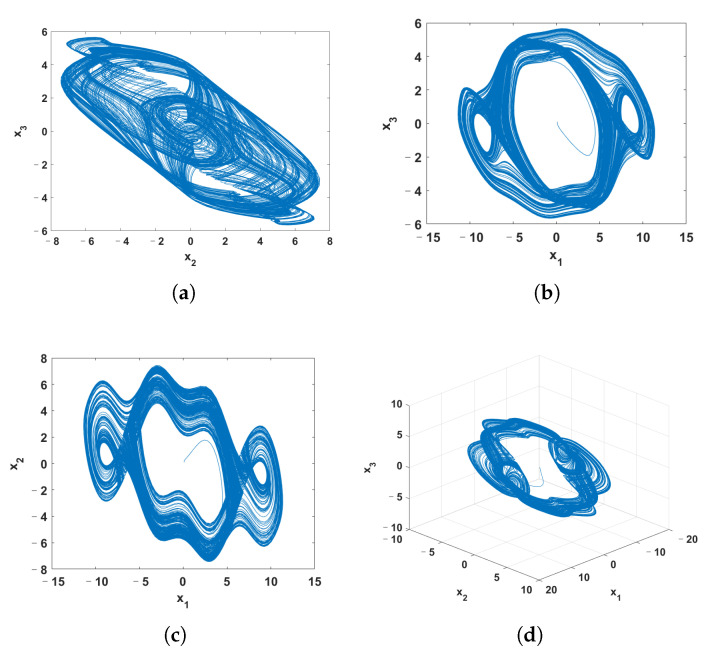
The phase portraits of system (4). (**a**) $x_{2}$-$x_{3}$ plane; (**b**) $x_{1}$-$x_{3}$ plane; (**c**) $x_{1}$-$x_{2}$ plane; (**d**) perspective view.

To confirm the chaotic characteristics of the system (4), we analyze its dynamic behavior. Figure 2 shows the relationship between the Lyapunov exponent (LE) of the system (4) and parameter *c*, where the LE is obtained by Euler method and Qatari Rial (QR) decomposition method. Figure 3 is the $x_{2}$-axis bifurcation diagram of the system (4). Figure 3 shows that the system (4) enters into chaos by period doubling bifurcation. Figure 4 shows the phase portraits of system (4) when *c* = 20.

**Figure 5:**
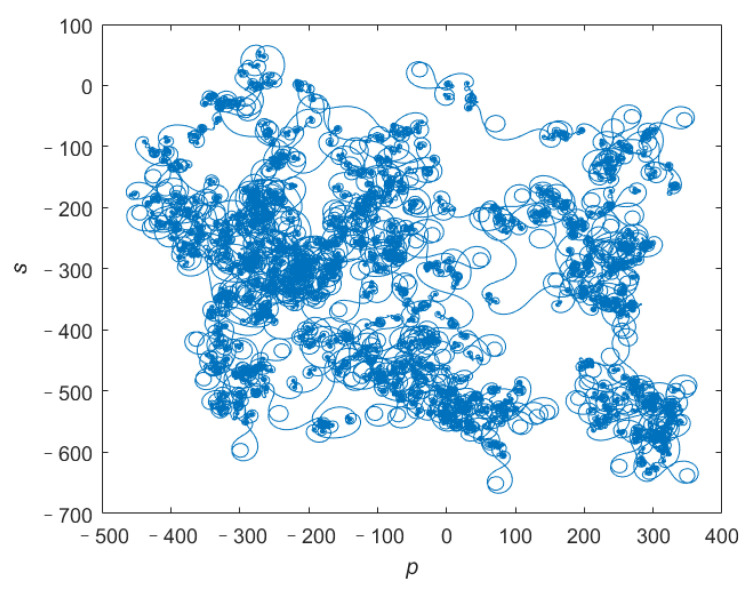
(*p*, *s*) plane of $x_{3}$ sequence with *c* = 20.

In addition, we use the 0–1 test [26] to further verify if the system (4) is chaotic. The trajectory of the (*p*, *s*) plane corresponds to Brownian motion when the parameter *c* of system (4) is 20, as shown in Figure 5. The trajectory of (*p*, *s*) plane of 0–1 test indicates that when parameter *c* is 20, the system (4) is chaotic.

### 2.2. Fractional Order CNN

Fractional calculus has a number of definitions, among which Caputo, Riemann-Liouville and Grunwald-Letnikov definitions are the most commonly used. Since the fractional order differential equation defined by Caputo definition has the same initial condition form as an integer order differential equation [17], Caputo definition is adopted in this paper. The definition of Caputo fractional calculus is

(5) $$D_{t}^{q}f\left(t\right)=\left\{\begin{array}{cc} \frac{1}{\Gamma (m-q)}\int_{0}^{t}\frac{f^{\left(m\right)}\left(\tau \right)}{{\left(t-\tau \right)}^{q+1-m}}d\tau & \mathrm{if}\;\;m-1<q<m;\\ \frac{d^{m}}{dt^{m}}f\left(t\right) & \mathrm{if}\;\;q=m, \end{array}\right.$$

where Γ(x) is Gamma function, which is

(6) $$\Gamma \left(x\right)=\int_{0}^{\infty }e^{-t}\cdot t^{x-1}dt.$$

To improve the randomness of sequences generated by chaotic system, we generalize the system (4) to fractional order case and propose the fractional order CNN, which is defined as

(7) $$\left\{\begin{array}{c} D_{t}^{q}x\left(t\right)=2y+2\tanh\left(x\right)+\tanh\left(y\right)-9\tanh\left(z\right);\\ D_{t}^{q}y\left(t\right)=z-9\tanh\left(x\right)+2\tanh\left(y\right)+4\tanh\left(z\right)+c\sin\left(x\right);\\ D_{t}^{q}z\left(t\right)=-3y-5z+\tanh\left(x\right)-9\tanh\left(y\right)+2\tanh\left(z\right). \end{array}\right.$$

**Figure 6:**
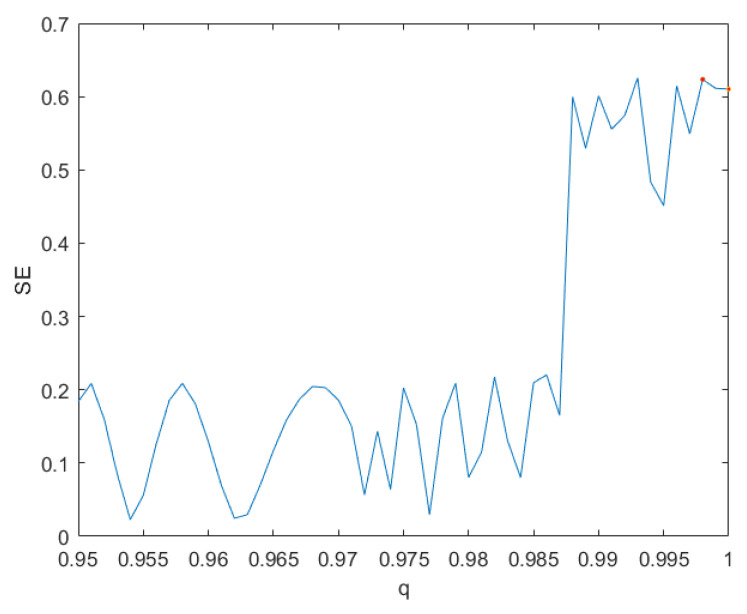
The complexity of system (7).

The complexity of chaotic systems refers to employing related algorithms to measure the possibility that the sequences generated by chaotic system approach random sequences [27]. The greater the complexity of chaotic system, the more random the sequences generated by chaotic systems are. Since spectral entropy (SE) algorithm [28] has the advantages of fewer parameters and higher accuracy, we use the SE algorithm to measure the complexity of the system (7) with parameter *c* = 20. Figure 6 shows the result, which illustrates that the complexity of system (7) with *q* = 0.998 and *q* = 1 (the system (4)) is 0.623 and 0.610, respectively. Obviously, the system (7) with *q* = 0.998 has higher complexity than the system (4).

**Figure 7:**
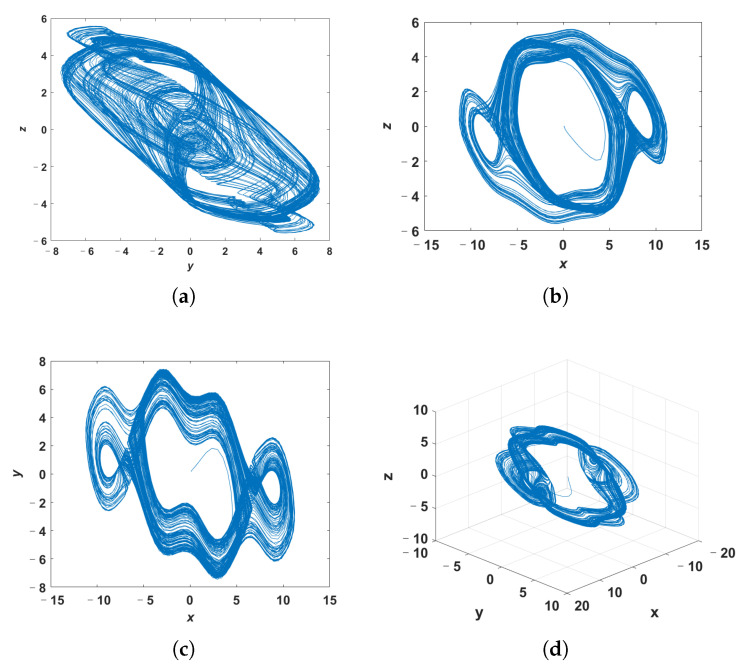
The phase portraits of system (7). (**a**) *y*-*z* plane; (**b**) *x*-*z* plane; (**c**) *x*-*y* plane; (**d**) perspective view.

**Figure 8:**
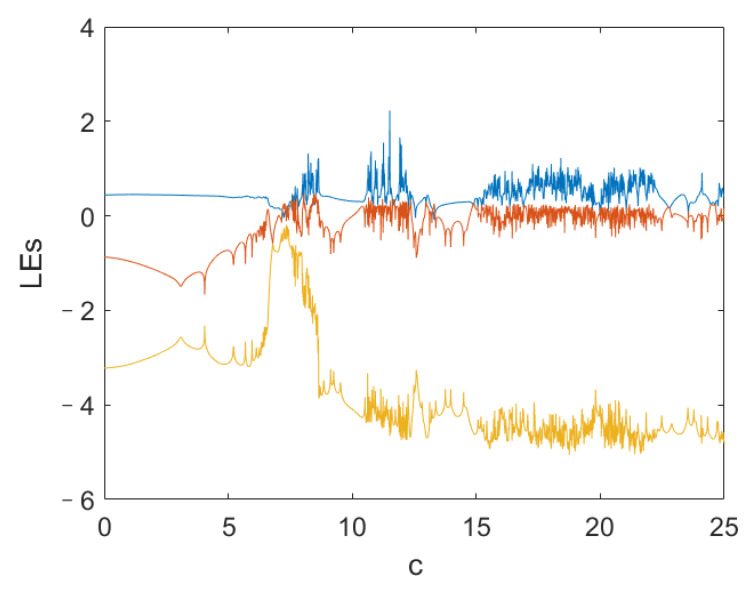
LE spectrum of the system (7).

Since the high complexity of the system does not mean that the system is chaotic, we perform dynamic analysis on the system (7) with *q* = 0.998 and *c* = 20 to determine whether it is chaotic or not. The phase portraits of system (7) with *q* = 0.998 and *c* = 20 are shown in Figure 7, in which the system (7) is solved by the predictor-corrector method. Figure 8 presents the LE of the system (7) calculated by Wolf’s method, where the order *q* is 0.998. According to the phase portraits and the LE, it can be judged that when the parameter *c* is 20 and the order *q* is 0.998, the system (7) is chaotic. Based on the above results, it can be seen that the system (7) with *q* = 0.998 and *c* = 20 is chaotic and has a higher complexity than the system (4).

In [25], an integer order CNN is proposed. We also generalize it to the fractional order case, which can be described as

(8) $$\left\{\begin{array}{c} D_{t}^{q}x\left(t\right)=2y+5\tanh\left(x\right)-\tanh\left(z\right);\\ D_{t}^{q}y\left(t\right)=z-6\tanh\left(x\right)+2\tanh\left(y\right)+4\tanh\left(z\right);\\ D_{t}^{q}z\left(t\right)=-3y-5z-\tanh\left(x\right)-3\tanh\left(y\right)+10\tanh\left(z\right)+20\sin\left(x\right). \end{array}\right.$$

**Figure 9:**
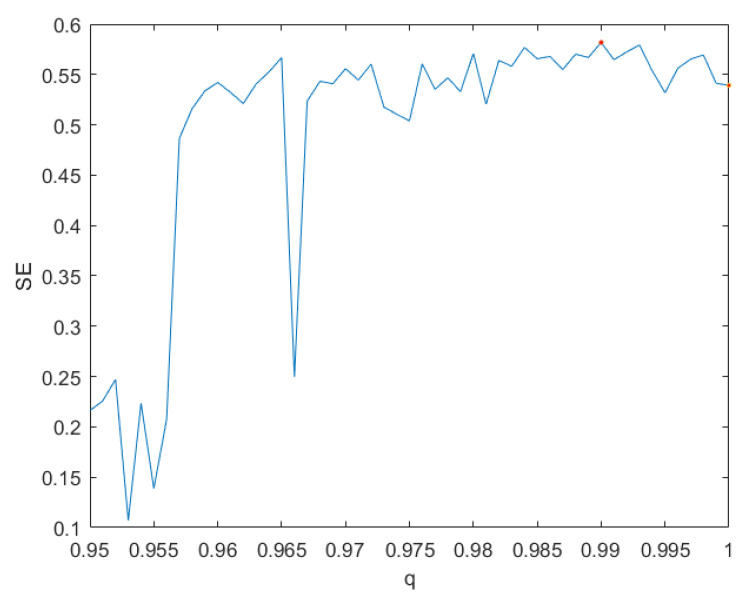
The complexity of system (8).

**Figure 10:**
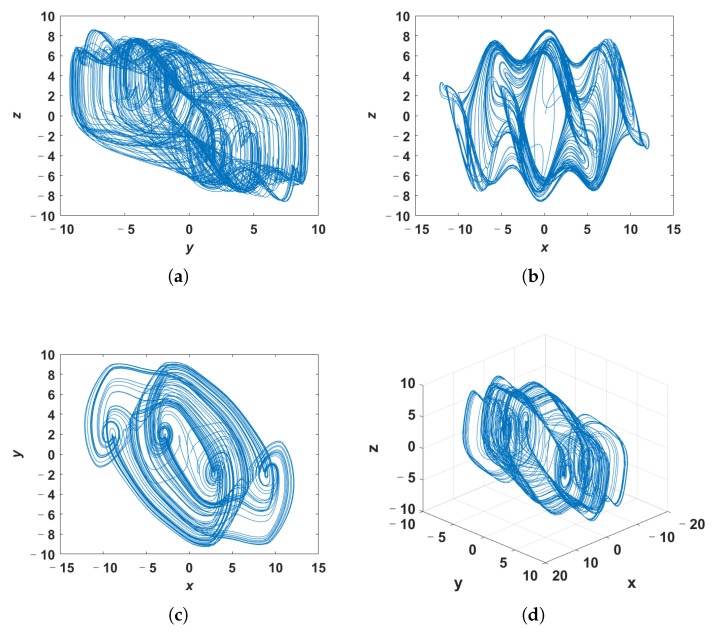
The phase portraits of system (8). (**a**) *y*-*z* plane; (**b**) *x*-*z* plane; (**c**) *x*-*y* plane; (**d**) perspective view.

According to the previous method, we conducted dynamic analysis on system (8). Figure 9 gives the SE of system (8) and illustrates that the complexity of the system (8) with *q* = 0.99 is the highest. Therefore, the order *q* of the system (8) is determined to be 0.99. Figure 10 gives the phase portraits of the system (8) with *q* = 0.99. In addition, the maximum LE of the system (8) with *q* = 0.99 solved by Wolf’s method is greater than 0. Therefore, system (8) with *q* = 0.99 is chaotic and has high complexity.

## 3. Fundamental Knowledge

### 3.1. Bit Plane Decomposition

An *n*-bit binary sequence can be used to represent any decimal value *b* that is not less than 0 [29], so an 8-bit binary sequence can be used to represent each pixel value of the image. In this paper, the three components of color image are decomposed into 8 bit planes respectively. The decomposition process is

(9) $$b=\sum_{j=1}^{8}c_{j}2^{j-1}=c_{8}\cdot 2^{7}+c_{7}\cdot 2^{6}+c_{6}\cdot 2^{5}+c_{5}\cdot 2^{4}+c_{4}\cdot 2^{3}+c_{3}\cdot 2^{2}+c_{2}\cdot 2^{1}+c_{1}\cdot 2^{0}.$$

### 3.2. DNA Sequence Operations

#### 3.2.1. DNA Encoding and Decoding Rules

A(adenine), G (guanine), C (cytosine), and T (thymine) are the four basic nucleic acids that make up each DNA sequence, where G and C, T and A are complementary respectively [30]. 0 and 1 are complementary in binary computation, so the binary array 11 and 00, 10 and 01 are complementary. Because binary arrays and DNA have similar complementary properties, the binary arrays 00, 11, 01, and 10 can be encoded as C, A, G and T. Watson and Crick found that among the 24 coding rules, only 8 coding rules meet the complement requirements, which are listed in Table 1.

Different from other encryption algorithms, this paper dynamically selects two encoding rules for the encryption algorithm. Assume that the pixel value is 39, which can be expressed as [00100111], and the selected coding rules are rule 1 and rule 6. In encryption, [00100111] is encoded as [ACGT] according to rule 1. Then, [ACGT] is decoded as [01110010] according to rule 6. In decryption, [01110010] is encoded as [ACGT] according to rule 6, then [ACGT] is decoded as [00100111] according to rule 1. Thus, the interlaced dynamic DNA encoding and decoding encryption scheme has the characteristic of evolution, which can make DNA operation more unpredictable and reduce the insecurity caused by the fixed encoding rule.

#### 3.2.2. DNA Operation

The DNA XOR operation is the XOR operation of binary number, so there are eight DNA XOR operations that correspond to DNA encoding rules. The DNA XOR operation that corresponds to encoding rule 2 is shown in Table 2.

### 3.3. Zigzag Confusion

**Figure 11:**
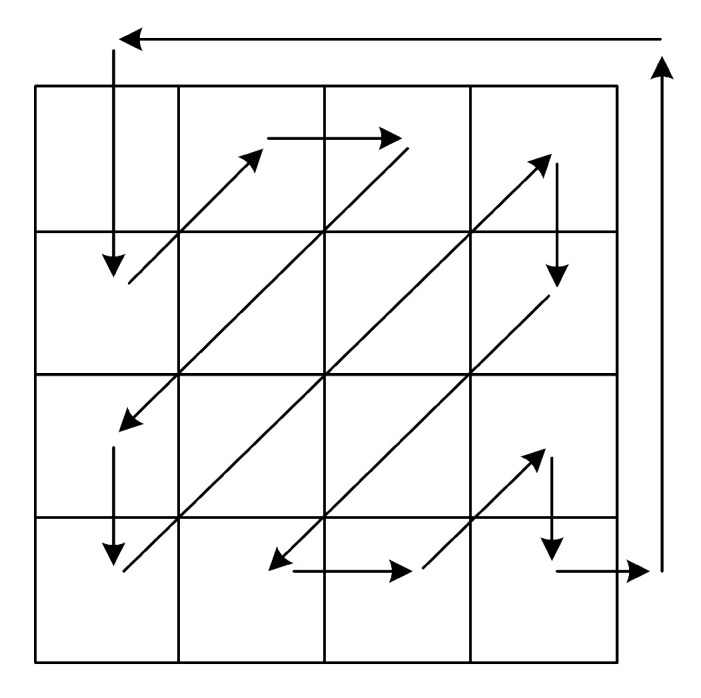
The path of the zigzag confusion.

**Figure 12:**
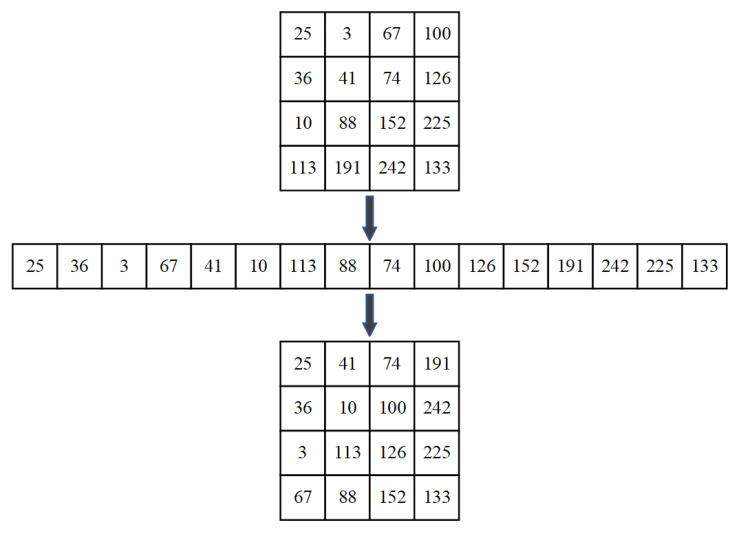
The example of zigzag confusion.

The path of the zigzag confusion is shown in Figure 11, which is different from the path of the general zigzag confusion. In this paper, zigzag confusion scans the elements in the matrix in Z order starting with the first element in the upper-left corner of the matrix, and rearranges the elements in columns into a matrix of the same size. In Figure 12, we give an example to help understand how the zigzag confusion works.

### 3.4. Convolution Operation

**Figure 13:**
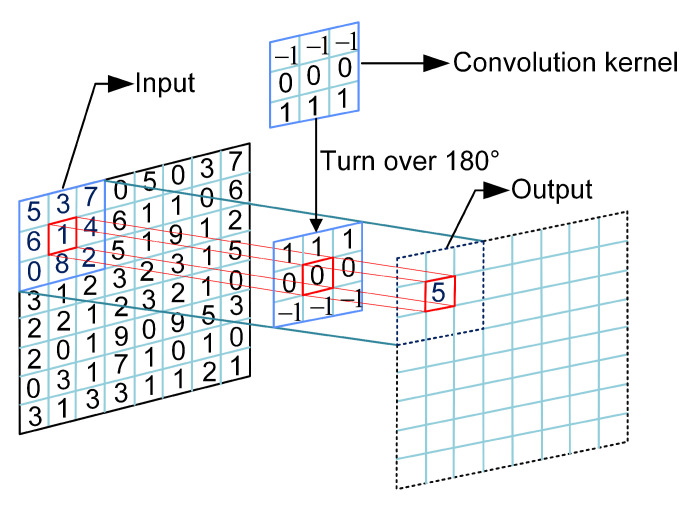
Convolution operation.

Convolution operation is widely used in the convolutional neural network, which is among the representative algorithms of deep learning and has excellent performance in computer vision, atmospheric science, natural language processing, and other fields. The definition of the convolution operation is

(10) $$h\left(x,y\right)=f\left(x,y\right)*g\left(x,y\right)=\sum_{i=-\infty }^{\infty }\sum_{j=-\infty }^{\infty }f(i,j)\cdot g(x-i,y-j),$$

where *h* represents the output, *f* represents the input, *g* represents the convolution kernel. Figure 13 shows how the convolution works. In this paper, the convolution operation is used to calculate plaintext index, in which the chaotic sequence is the input and the hash value of plain image is the convolution kernel.

## 4. The Proposed Image Encryption and Decryption Algorithm

### 4.1. Generating the Chaotic Matrices

The proposed fractional-order CNNs are used to generate chaotic matrices, and the following is the specific generating procedure for chaotic matrices.

**Step 1:** From the input color plain image *P* of size M×N, a 256-bit hash value *K* is generated by using the secure hash algorithm (SHA-256), and *K* is converted to 32 numbers $k_{1},k_{2},\cdots ,k_{32}$ with every 8 bits as a group.

**Step 2:** Calculate the initial values of systems (7) and (8), as illustrated in Algorithm 1.
**Algorithm 1** Generating initial values of fractional order CNNs.**Input:** $k_{1},k_{2},\cdots ,k_{32}$ 1: $x_{0}=\mathrm{mod}(k_{1}⊕k_{17}+\sum_{i=1}^{11}k_{3i-1},256)/2^{8}$; 2: $y_{0}=\mathrm{mod}(k_{2}⊕k_{18}+\sum_{i=1}^{10}k_{3i},256)/2^{8}$; 3: $z_{0}=\mathrm{mod}(k_{3}⊕k_{19}+\sum_{i=0}^{10}k_{3i+1},256)/2^{8}$;  where ⊕ represents XOR operation.**Output:** $x_{0},y_{0},z_{0}$.

**Step 3:** Firstly, perform 2000 pre-iterations for the systems (7) and (8) to avoid transient effects. Then, the systems (7) and (8) are iterated $\left\lceil M\times N÷3\right\rceil$ times respectively, where $\left\lceil b\right\rceil$ represents the nearest integer greater than or equal to *b*. The *X*, *Y* and *Z* sequences generated by the system (7) and the system (8) are spliced into $D_{1}$ and $D_{2}$ respectively (*X* followed by *Y*, and *Y* followed by *Z*). Finally, $Z_{1}$ and $Z_{2}$ are the first M×N data of $D_{1}$ and $D_{2}$ respectively.

**Step 4:** Chaotic matrices $X_{1}$, $Y_{1}$, $X_{2}$, $Y_{2}$, $X_{3}$, $Y_{3}$, $X_{4}$ and $Y_{4}$ of size M×N are generated by

(11) $$\left\{\begin{array}{c} X_{1}\left(i,j\right)=\mathrm{mod}(\mathrm{floor}(Z_{1}((i-1)\cdot N+j)\cdot 10^{14}),256);\\ Y_{1}\left(i,j\right)=\mathrm{mod}(\mathrm{floor}(Z_{2}((i-1)\cdot N+j)\cdot 10^{14}),256);\\ X_{2}\left(i,j\right)=\mathrm{mod}(\mathrm{floor}(Z_{1}((i-1)\cdot N+j)\cdot 10^{13}),256);\\ Y_{2}\left(i,j\right)=\mathrm{mod}(\mathrm{floor}(Z_{2}((i-1)\cdot N+j)\cdot 10^{13}),256);\\ X_{3}\left(i,j\right)=\mathrm{mod}(\mathrm{floor}(Z_{1}((i-1)\cdot N+j)\cdot 10^{12}),256);\\ Y_{3}\left(i,j\right)=\mathrm{mod}(\mathrm{floor}(Z_{2}((i-1)\cdot N+j)\cdot 10^{12}),256);\\ X_{4}\left(i,j\right)=\mathrm{floor}(Z_{1}((i-1)\cdot N+j)\cdot 10^{11});\\ Y_{4}\left(i,j\right)=\mathrm{floor}(Z_{2}((i-1)\cdot N+j)\cdot 10^{11}). \end{array}\right.$$

### 4.2. Forward/Backward Bit-Level Diffusion

In the process of forward bit-level diffusion, we first XOR the lowest bit plane of image with the lowest bit plane of chaotic matrix, and then diffuse one by one from the lowest bit plane to the highest bit plane. In the process of backward bit-level diffusion, we first XOR the highest bit plane of image with the highest bit plane of chaotic matrix, and then diffuse them one by one from the highest bit plane to the lowest bit plane. Suppose *Q* is any component of color image and *R* is chaotic matrix. The following is the specific forward bit-level diffusion process.

**Step 1:** The *Q* and *R* are decomposed into 8 bit planes: $Q_{1}$, $Q_{2}$, $Q_{3}$, $Q_{4}$, $Q_{5}$, $Q_{6}$, $Q_{7}$, $Q_{8}$, $R_{1}$, $R_{2}$, $R_{3}$, $R_{4}$, $R_{5}$, $R_{6}$, $R_{7}$ and $R_{8}$.

**Step 2:** The 8 bit planes of *Q* and *R* are turned into DNA matrices according to DNA encoding rule $q_{1}$: $Q_{12\_DNA}$, $Q_{34\_DNA}$, $Q_{56\_DNA}$, $Q_{78\_DNA}$, $R_{12\_DNA}$, $R_{34\_DNA}$, $R_{56\_DNA}$ and $R_{78\_DNA}$.

**Step 3:** The DNA matrices of *Q* are diffused through

(12) $$\left\{\begin{array}{c} Q_{12\_DNA}^{{}^{\prime }}=Q_{12\_DNA}⊕R_{12\_DNA};\\ Q_{34\_DNA}^{{}^{\prime }}=\left(Q_{34\_DNA}⊕R_{34\_DNA}\right)⊕Q_{12\_DNA}^{{}^{\prime }};\\ Q_{56\_DNA}^{{}^{\prime }}=\left(Q_{56\_DNA}⊕R_{56\_DNA}\right)⊕Q_{34\_DNA}^{{}^{\prime }};\\ Q_{78\_DNA}^{{}^{\prime }}=\left(Q_{78\_DNA}⊕R_{78\_DNA}\right)⊕Q_{56\_DNA}^{{}^{\prime }}, \end{array}\right.$$

where ⊕ represents DNA XOR operation corresponding to encoding rule $q_{1}$.

**Step 4:** DNA matrix $Q^{\prime }$ is decoded according to DNA decoding rule $q_{2}$.

The backward bit-level diffusion can be obtained by replacing Equation (12) in forward bit-level diffusion with

(13) $$\left\{\begin{array}{c} Q_{78\_DNA}^{{}^{\prime }}=Q_{78\_DNA}⊕R_{78\_DNA};\\ Q_{56\_DNA}^{{}^{\prime }}=\left(Q_{56\_DNA}⊕R_{56\_DNA}\right)⊕Q_{78\_DNA}^{{}^{\prime }};\\ Q_{34\_DNA}^{{}^{\prime }}=\left(Q_{34\_DNA}⊕R_{34\_DNA}\right)⊕Q_{56\_DNA}^{{}^{\prime }};\\ Q_{12\_DNA}^{{}^{\prime }}=\left(Q_{12\_DNA}⊕R_{12\_DNA}\right)⊕Q_{34\_DNA}^{{}^{\prime }}, \end{array}\right.$$

where ⊕ represents DNA XOR operation corresponding to encoding rule $q_{1}$.

### 4.3. Plaintext Associative Permutation

The process of plaintext associative permutation is as follows.

**Step 1:** The *K* is reshaped by column into a 16×16 matrix, and the matrix is used as the convolution kernel to convolute with the chaotic matrix $Y_{4}$.

**Step 2:** Perform modular M×N operation on the output of convolution operation to obtain matrix *S*, where *M* and *N* denote the size of the image *I* after forward bit-level diffusion.

**Step 3:** Arrange the elements that do not appear in *S* in the order of large to small to get sequence *T*.

**Step 4:** Scramble *T* with $X_{4}$ as the index.

**Step 5:** Replace the repeated elements in *S* with the elements in *T*, and then rearrange the image *I* with *S* as the index.

The operation details are given in Algorithm 2.
**Algorithm 2** The plaintext associative permutation.**Input:** The image *I*, 256-bit hash value *K*, chaotic matrices $X_{4}$ and $Y_{4}$. 1:*K* is reshaped into a matrix. 2:Get the number of rows *M* and columns *N* of the image *I*. 3:Convolute $Y_{4}$ with *K*, and store the convolution result in *S*. 4:S=mod(S,M×N)+1; 5:$T=\mathrm{sort}(\mathrm{setdiff}(1:M\times N,S){,}^{\prime }{\mathrm{descend}}^{\prime })$; 6:$X_{4}=\mathrm{mod}(X_{4}(1:\mathrm{length}(T)),\mathrm{length}(T))+1$; 7:**for** i=1 to length(T) **do** 8:    e=T(i); 9:    $T\left(i\right)=T(X_{4}\left(i\right))$; 10:    $T(X_{4}\left(i\right))=e$ 11:**end for** 12:Get *A* and *B*. *A* is the same data as in *S*, but with no repetitions. *B* is the index vectors of *A* in *S*. 13:E=setdiff(1:M×N,B); 14:**for** i=1 to length(E) **do** 15:    S(E(i))=T(i); 16:**end for** 17:**for** i=1 to M×N **do** 18:    $I{}^{\prime }(S(i))=I(i)$; 19:**end for**where setdiff(1:M×N,S) returns the data in 1:M×N that is not in *S*.**Output:** $I{}^{\prime }$.


### 4.4. The Complete Encryption Process

**Figure 14:**
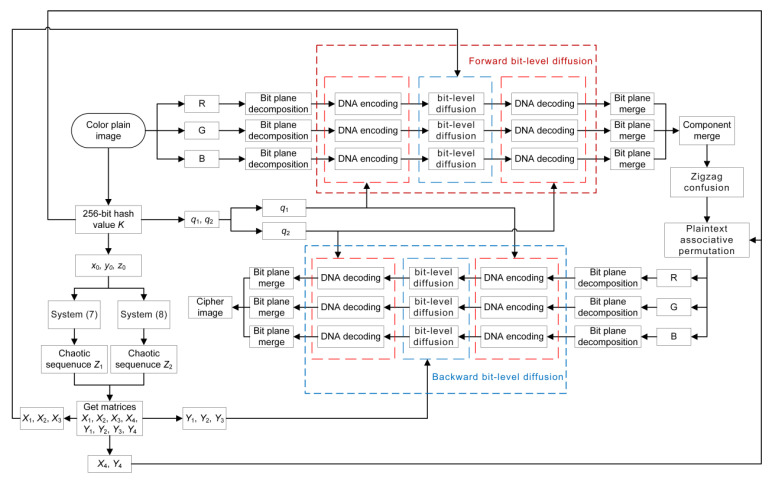
The encryption flow chart.

Figure 14 illustrates the encryption flow chart of the proposed algorithm. The following is the specific steps.

**Step 1:** Input a color plain image and generate the key *K* and the chaotic matrices, as described in Section 4.1.

**Step 2:** Calculate $q_{1}$ and $q_{2}$ by

(14) $$\left\{\begin{array}{c} q_{1}=\;\mathrm{mod}\;(\sum_{i=1}^{128}K(i),8)+1;\\ q_{2}=\;\mathrm{mod}\;(\sum_{i=129}^{256}K(i),8)+1. \end{array}\right.$$

**Step 3:** The R, G, B components of the color image are decomposed into 8 bit planes, respectively.

**Step 4:** Chaotic matrices $X_{1}$, $X_{2}$ and $X_{3}$ are used to perform forward bit-level diffusion on each component of the image, as described in Section 4.2.

**Step 5:** The bit planes of each component are merged, and then the three components of the image are merged.

**Step 6:** Perform zigzag confusion on the image as shown in Section 3.3, and then plaintext associative permutation is performed on the image as illustrated in Section 4.3.

**Step 7:** Repeat step 3, and then chaotic matrices $Y_{1}$, $Y_{2}$ and $Y_{3}$ are used to perform backward bit-level diffusion on each component of the image, as described in Section 4.2.

**Step 8:** Obtain the cipher image by repeating step 5.

**Figure 15:**
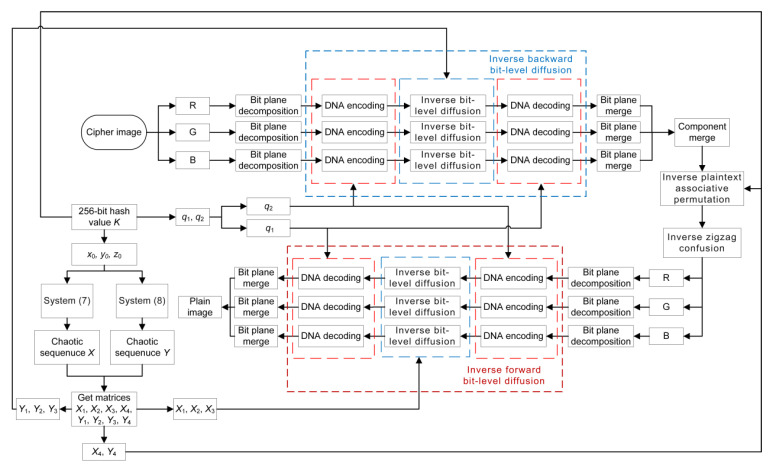
The decryption flow chart.

The decryption algorithm can be obtained by reverse operation of the encryption algorithm. Figure 15 shows the decryption algorithm flow chart.

## 5. Simulation Results

**Figure 16:**
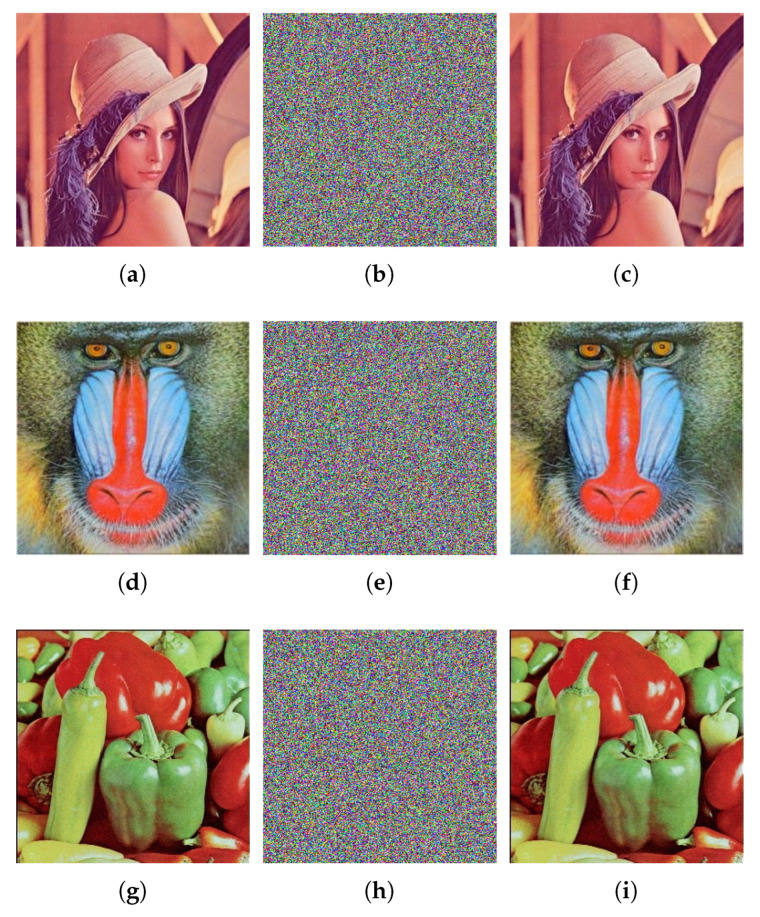
Encrypted and decrypted images. (**a**) Lena; (**b**) encrypted image of Lena; (**c**) decrypted image of Lena; (**d**) Baboon; (**e**) encrypted image of Baboon; (**f**) decrypted image of Baboon; (**g**) Pepper; (**h**) encrypted image of Pepper; (**i**) decrypted image of Pepper.

Figure 16 illustrates the simulation results. Obviously, the cipher images are like noise and cannot be recognizable. This means that, even if cipher images are intercepted in transit, valid information about plain images will not be leaked. In addition, the images decrypted by the correct key are visually identical to corresponding plain images. To quantitatively evaluate the quality of the decrypted images of the proposed algorithm, we introduce the Peak Signal-to-Noise Ratio (PSNR), which is defined as

(15) $$\mathrm{PSNR}=20\cdot \log_{10}\left(\frac{255}{\sqrt{\frac{1}{M\cdot N}\cdot \sum_{i=1}^{M}\sum_{j=1}^{N}{\left(D\left(i,j\right)-P\left(i,j\right)\right)}^{2}}}\right),$$

where *D* and *P* represent the decrypted image and the plain image respectively, (*i*, *j*) are the position of pixel, *M* and *N* are the size of the images. The larger the PSNR value between the plain image and the decrypted image, the smaller the difference between them. When the decrypted image is completely the same as the plain image, the denominator in Equation (15) is 0, and the value of PSNR is infinity (Inf). The test results are shown in Table 3. It can be seen that the PSNR values between the decrypted image and the plain image are infinity. This indicates that the decrypted images are completely the same as the corresponding plain images. Therefore, the proposed algorithm performs well in terms of encryption and decryption.

## 6. Security Analyses

This section analyzes the following indicators to demonstrate the proposed algorithm’s security performance: key space, histogram, correlation of adjacent pixels, key sensitivity, differential attack, chosen/known-plaintext attack, information entropy, occlusion attack, and noise attack.

### 6.1. Key Space Analysis

Image encryption algorithms with key space smaller than $2^{100}$ are considered insecure [31]. The proposed algorithm’s key is composed of 256-bit binary hash values, and its key space size is $2^{256}$, which is greater than $2^{100}$. Consequently, the proposed algorithm can defend violent attacks.

### 6.2. Histogram Analysis

**Figure 17:**
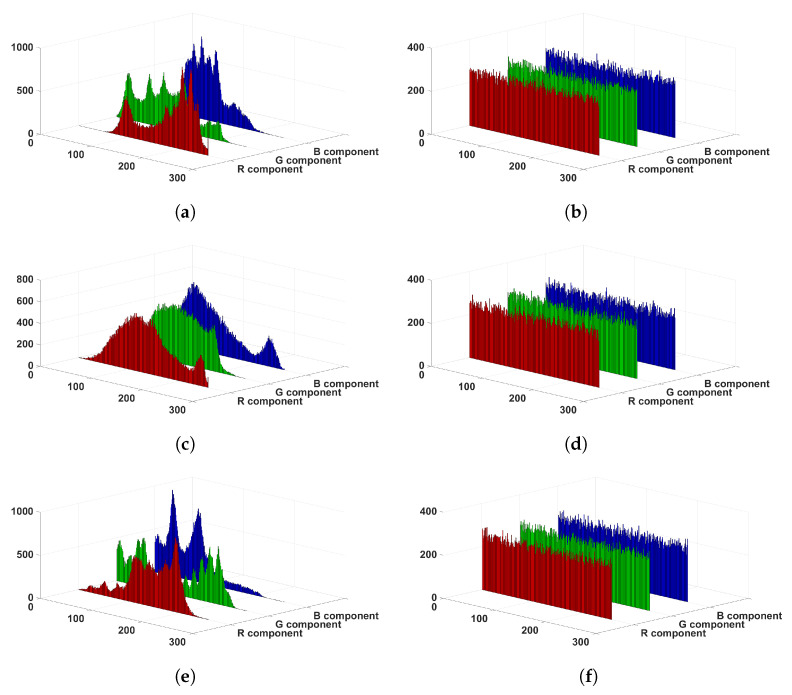
Histogram results. (**a**) Lena; (**b**) cipher image of Lena; (**c**) Baboon; (**d**) cipher image of Baboon; (**e**) Pepper; (**f**) cipher image of Pepper.

A histogram can provide an intuitive insight into the distribution characteristics of image pixel values, so we give the histograms of images, as shown in Figure 17. In contrast to the plain images, the pixel values of cipher images are distributed uniformly. This means that the attacker will not be able to obtain the information from the plain image via statistical analysis attack. In addition, to further examine the uniformity of histograms, chi-square test is introduced. It is defined as

(16) $$\chi^{2}=\sum_{i=0}^{255}\frac{{\left(f_{i}-g\right)}^{2}}{g},$$

where g=M×N/256, and $f_{i}$ is the occurrence frequency of the pixel with the value of *i*. When the chi-square value is smaller than 293.2478, it means that the image histogram is approximately evenly distributed at the confidence level of 0.05 [32]. Table 4 shows that the cipher images’ chi-square values are all smaller than 293.2478, so the cipher images’ histograms are approximately evenly distributed. As a result, the proposed algorithm is capable of invalidating statistical attacks.

### 6.3. Correlation Analysis of Adjacent Pixels

The correlation between adjacent pixels is closely related to whether the cipher image will be broken by statistical attack. The correlation between adjacent elements of cipher image should be as low as possible to prevent the cipher image from statistical attacks. In order to make the analysis result more reliable, 20,000 pairs of pixels are chosen at random and correlation coefficients are calculated through

(17) $$\left\{\begin{array}{c} r_{xy}=\frac{cov(x,y)}{\sqrt{D(x)}\cdot \sqrt{D(y)}};\\ D\left(x\right)=\frac{1}{N}\cdot \sum_{i=1}^{N}{\left(x_{i}-E\left(x\right)\right)}^{2};\\ cov\left(x,y\right)=\frac{1}{N}\cdot \sum_{i=1}^{N}\left(x_{i}-E\left(x\right)\right)\cdot \left(y_{i}-E\left(y\right)\right);\\ E\left(x\right)=\frac{1}{N}\cdot \sum_{i=1}^{N}x_{i}, \end{array}\right.$$

where $x_{i}$ and $y_{i}$ are the gray values of the pixels and *N* is the number of pixel pairings that have been chosen.

**Figure 18:**
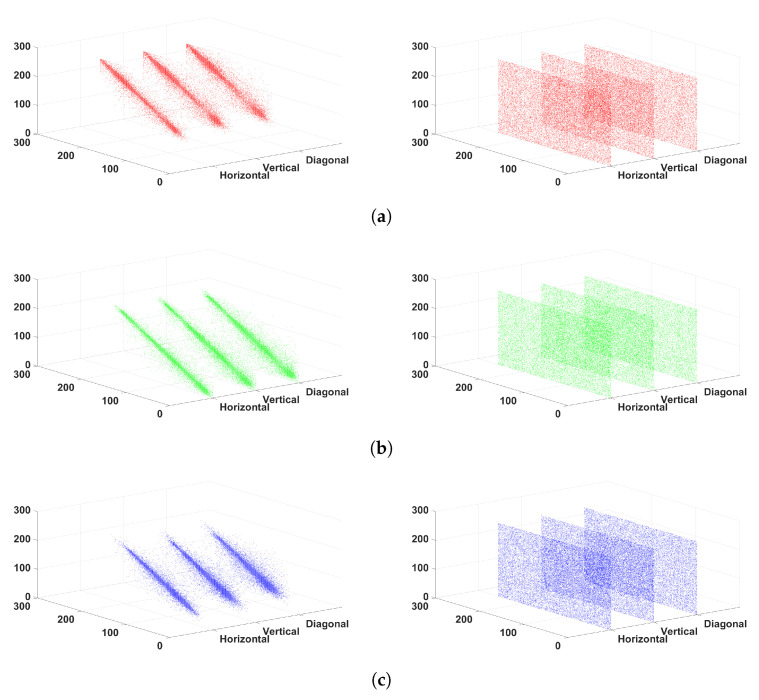
Correlation scatterplots of Lena image with and without encryption. (**a**) R component; (**b**) G component; (**c**) B component.

Before and after encryption, the correlation coefficient between adjacent pixels clearly changes, and the cipher image’s correlation coefficients of adjacent pixels are near to 0, as seen in Table 5. Figure 18 displays the correlation scatterplots of the Lena image with and without encryption, where the left column is the correlation scatterplots of plain image, and the right column is the correlation scatterplots of cipher image. The adjacent pixel pairs in cipher images are evenly distributed in different components and directions, unlike in plain images.

The comparisons between the proposed algorithm and other image encryption algorithms are placed in Table 6. By analyzing Table 6, it can be obtained that, as a whole, the proposed algorithm has smaller correlation coefficients compared with Refs. [33,34,35,36,37].

### 6.4. Key Sensitivity Analysis

The key sensitivity of encryption algorithm is a vital metric to evaluate its security. Sensitivity of the key will be examined from two perspectives: the encryption and decryption process. The key *K* is obtained by performing SHA-256 on plain image, and the new key $K_{1}$ is obtained by randomly changing one bit of *K* using

(18) K(i)=mod(K(i)+1,2).

**Figure 19:**
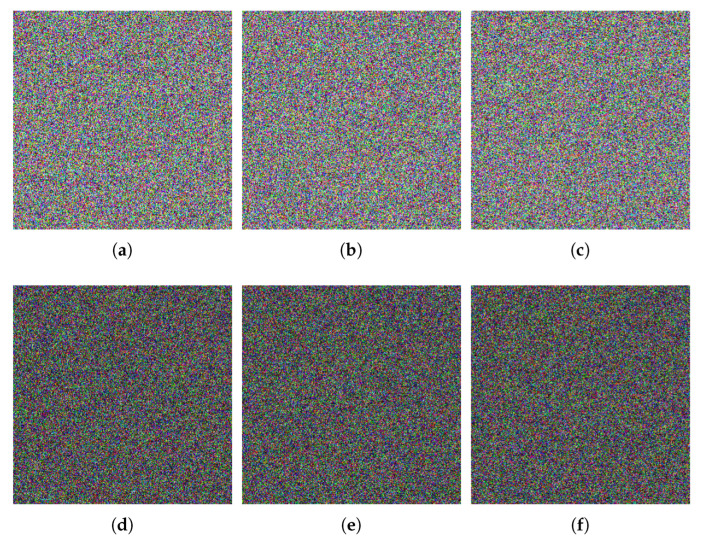
Key sensitivity test results during encryption. (**a**) Lena’s cipher image encrypted by $K_{1}$; (**b**) Baboon’s cipher image encrypted by $K_{1}$; (**c**) Pepper’s cipher image encrypted by $K_{1}$; (**d**) absolute value of (**a**) minus Figure 16b; (**e**) absolute value of (**b**) minus Figure 16e; (**f**) absolute value of (**c**) minus Figure 16h.

In encryption process, encrypting the identical image with *K* and $K_{1}$ obtains two cipher images. Figure 19 shows the experimental results. Obviously, the subtraction images are noise-like images. Therefore, the cipher images encrypted with $K_{1}$ are not the same as the cipher images encrypted with *K*. Moreover, we introduce the number of pixels change rate (NPCR) and uniform average change intensity (UACI) to quantitatively analyze the differences between the two cipher images. The NPCR and UACI are defined as

(19) $$\left\{\begin{array}{c} \mathrm{NPCR}=\sum_{i=1}^{M}\sum_{j=1}^{N}\frac{D(i,j)}{M\cdot N}\cdot 100\%;\\ \mathrm{UACI}=\sum_{i=1}^{M}\sum_{j=1}^{N}\frac{\left|C_{1}\left(i,j\right)-C_{2}\left(i,j\right)\right|}{255\cdot M\cdot N}\cdot 100\%. \end{array}\right.$$

According to Ref. [32], the ideal values of UACI and NPCR for two random 8-bit images are 33.4635% and 99.6094%, respectively. Clearly, the NPCR and UACI values are both near to the ideal values, as shown in Table 7.

**Figure 20:**
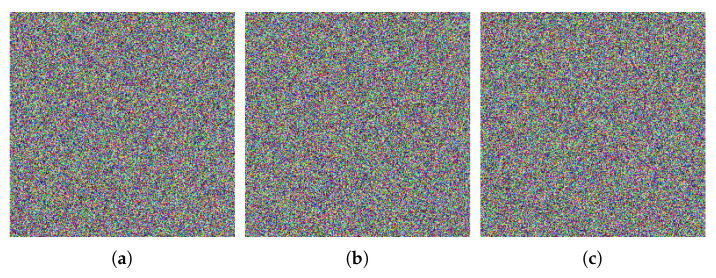
Experimental results of key sensitivity test during decryption. (**a**) result of decrypting Lena cipher image (Figure 16b) using $K_{1}$; (**b**) result of decrypting Baboon cipher image (Figure 16e) using $K_{1}$; (**c**) result of decrypting Pepper cipher image (Figure 16h) using $K_{1}$.

In decryption process, we decrypt the cipher image *C* with $K_{1}$ to obtain the image $P_{1}$, where the cipher image *C* is obtained by encrypting the plain image *P* with *K*. Figure 20 shows the results. As can be seen from Figure 20, the image decrypted with the wrong key cannot get any effective information through vision. Meanwhile, NPCR and UACI are again employed to quantify the differences between *P* and $P_{1}$. It is worth noting that the NPCR ideal value between deterministic and random images is fixed with a value of 99.6094%, while the UACI ideal value is dynamic [32]. When the Lena image (Figure 16a) is the deterministic image, 32.6967%, 30.5401% and 27.7562% are UACI ideal values of three components. When the Baboon image (Figure 16d) is the deterministic image, 29.4993%, 27.8160%, and 30.4805% are UACI ideal values of three components. When the Pepper image (Figure 16g) is the deterministic image, 28.7532%, 33.4662%, and 34.0153% are UACI ideal values of three components. Table 8 shows the NPCR and UACI between *P* and $P_{1}$. Table 8 indicates that the values of NPCR and UACI are relatively near to the corresponding ideal values, so the proposed algorithm has strong sensitivity to the key.

### 6.5. Information Entropy Analysis

The randomness of image information can be reflected through information entropy. The greater the information entropy, the less visual information the image contains, and the better the randomness of the image. The information entropy of information source *s* is defined as

(20) $$H\left(s\right)=\sum_{i=0}^{2^{m}-1}p(s_{i})\log\frac{1}{p(s_{i})},$$

where m=8, $s_{i}$ is the grayscale value, and $p(s_{i})$ represents the occurrence probability of $s_{i}$.

The theoretical value of information entropy of an 8-bit truly random image is 8. Table 9 illustrates the information entropy of the image before and after encryption. Clearly, the information entropy of the cipher images all distinctly approximate the theoretical value. Meanwhile, Table 10 gives the comparison results between the proposed algorithm and other algorithms on Lena image. As we can see, the information entropy of the proposed algorithm are higher than that of Refs. [34,35], and the proposed algorithm has some merits compared with Refs. [33,36,37].

### 6.6. Differential Attack Analysis

A secure image encryption algorithm can make the cipher image change dramatically when the plain image changes slightly. To verify the resistance of differential attacks of the proposed algorithm, we first select a pixel from the plain image $P_{1}$ at random and modify its value by Equation (21) to obtain the image $P_{2}$. Then, the cipher images $C_{1}$ and $C_{2}$ are obtained by encrypting images $P_{1}$ and $P_{2}$ using the proposed algorithm. Finally, the difference between $C_{1}$ and $C_{2}$ is quantified by NPCR and UACI. Table 11 gives the results of differential attack. The results noted that NPCR and UACI are close to ideal values, so the proposed algorithm can effectively spread the small differences of the color plain image to the cipher image.

(21) value=mod(value+1,256).

Table 12 presents the comparison between the proposed algorithm and other algorithms on the Lena image. By analyzing the data in Table 12, it can be obtained that the NPCR and UACI of the proposed algorithm are closer to the ideal value compared with Refs. [33,35,36]. Compared with Ref. [34], the NPCR of R and B components of the proposed algorithm are closer to the ideal value, and the UACI of R and G components of the proposed algorithm are closer to the ideal value. Therefore, the proposed algorithm has some advantages compared with Refs. [33,34,35,36].

### 6.7. Chosen/Known-Plaintext Attack Analysis

The common methods for breaking image encryption algorithms include the chosen-plaintext attack and the known-plaintext attack. Moreover, the image encryption algorithm which can withstand the chosen-plaintext attack can also withstand the known-plaintext attack [38]. Therefore, we only test the performance of the proposed algorithm against the chosen-plaintext attack.

**Figure 21:**
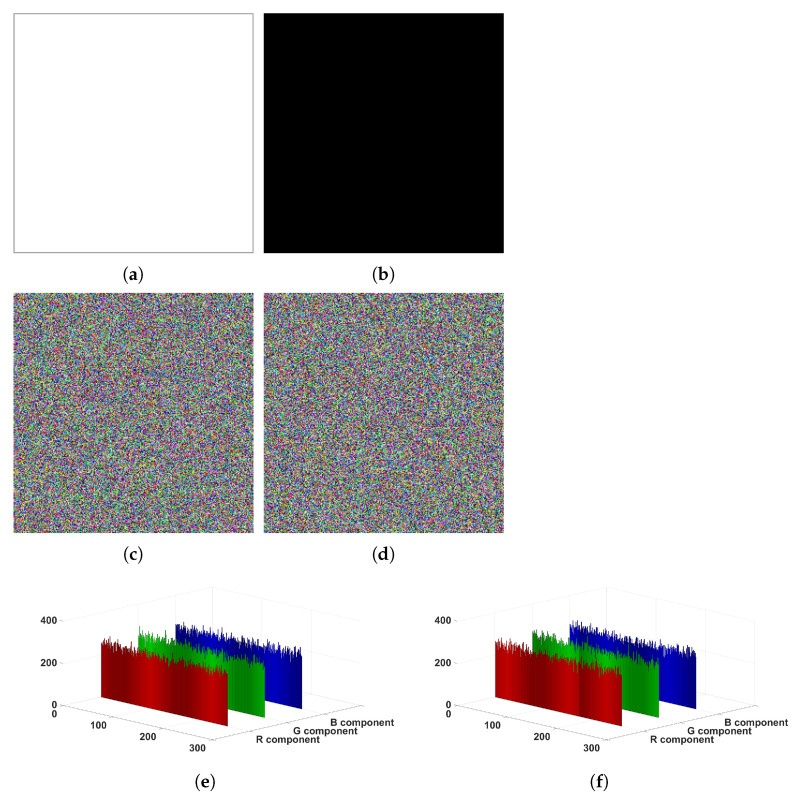
Experimental results of all-white image and all-black image. (**a**) all-white image; (**b**) all-black image; (**c**) cipher image of all-white image; (**d**) cipher image of all-black image; (**e**) histogram of (**c**); (**f**) histogram of (**d**).

Because all-white and all-black images can make the permutation process invalid, attackers often use them to break encryption algorithms. Here, we encrypt the all-white image and all-black image, respectively, and perform a series of analyses on the encrypted images. Figure 21 and Table 13 show the experimental results. Clearly, the cipher images of all-white and all-black are unrecognizable noise images and their pixel values are evenly distributed, as shown in Figure 21. Table 13 illustrates that the cipher images of all-white and all-black have good performance. Therefore, the proposed algorithm can effectively withstand both chosen-plaintext and known-plaintext attacks.

### 6.8. Occlusion Attack Analysis

**Figure 22:**
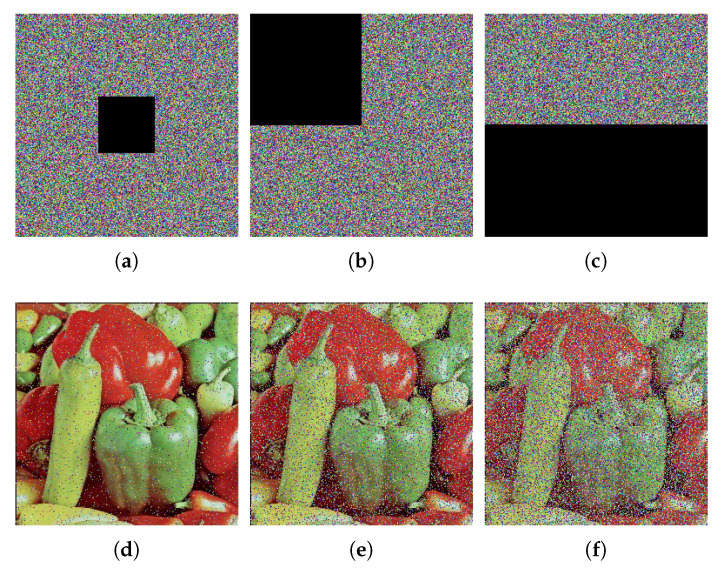
Occlusion attack results. (**a**) Figure 16h with 6.25% content occluding; (**b**) Figure 16h with 25% content occluding; (**c**) Figure 16h with 50% content occluding; (**d**) decrypted image of (**a**); (**e**) decrypted image of (**b**); (**f**) decrypted image of (**c**).

An effective image encryption algorithm should be robust to occlusion attack. Here, the color image Pepper (Figure 16g) is used as the test image. Furthermore, the content of the cipher image (Figure 16h) is occluded by 1/16, 1/4 and 1/2 respectively. Figure 22 gives the decryption results of the occluded images. clearly, the decrypted images can still be visually recognized even though the occlusion attack results in content loss and makes the decrypted image blurred. Therefore, the proposed algorithm is robust to occlusion attack.

### 6.9. Noise Attack Analysis

**Figure 23:**
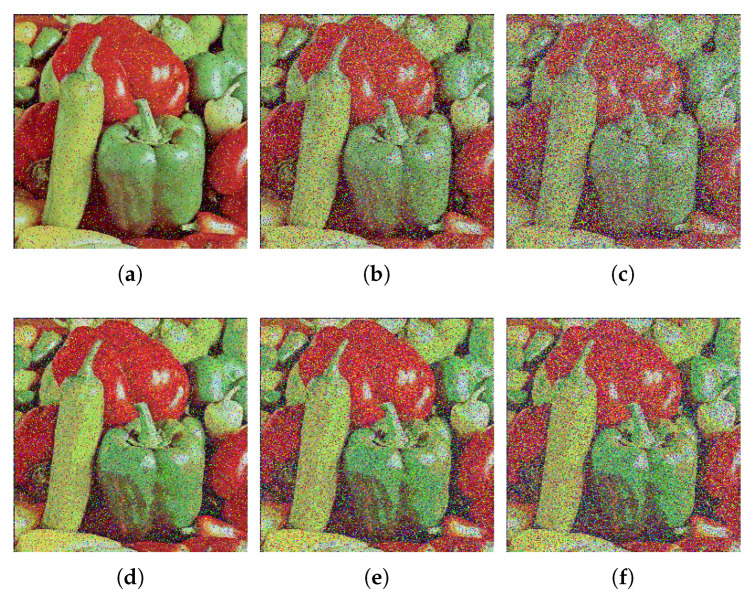
Decryption images under different noise and different intensity. (**a**) SPN of 0.1 intensity; (**b**) SPN of 0.3 intensity; (**c**) SPN of 0.5 intensity; (**d**) GN of variance 0.0001 and mean 0.01; (**e**) GN of variance 0.0005 and mean 0.01; (**f**) GN of variance 0.001 and mean 0.01.

Image is often disrupted by noise during transmission. To ensure the effective restoration of the cipher image, the proposed algorithm should have good anti-noise interference ability. Salt & pepper noise (SPN) and Gaussian noise (GN) with different intensity are utilized to test the anti-noise performance of the proposed algorithm. Figure 23 gives the test results. The cipher images disturbed by noise are still visually identifiable after decryption, as shown in Figure 23. As a result, the proposed algorithm has strong ability to resist the attack from noise.

## 7. Conclusions

In this paper, two fractional order CNNs have been proposed by using Caputo definition. According to the results of dynamic analysis, the proposed two fractional order CNNs had better chaotic characteristics. Meanwhile, a color image encryption algorithm based on double fractional order CNN, interlaced dynamic DNA encoding and decoding, zigzag confusion, bidirectional bit-level diffusion and convolution operation was proposed. Firstly, the proposed algorithm adopted the encryption structure of forward diffusion, permutation and backward diffusion. The encryption structure can prevent the chosen-plaintext attack from breaking the permutation process. Secondly, the diffusion process of the proposed algorithm was carried out on the bit plane, which can better hide the bit plane information of plain image. Moreover, the proposed algorithm adopted the interlaced dynamic DNA encoding and decoding rule (the selection of rule was related to plaintext), which can make the diffusion process have dynamic evolution characteristics. Finally, the permutation process of the proposed algorithm included two parts: zigzag confusion and plaintext association permutation. In the process of zigzag confusion, the scanning path was different from other algorithms. In process of plaintext associative permutation, the convolution operation was used to make the proposed algorithm more sensitive to the key. In addition, the hash value of the plain image was the key, so the proposed algorithm was highly correlated with the plain image. Simulation results and security analysis indicated that the proposed algorithm was secure and effective.

However, since the proposed algorithm adopted the fractional order chaotic system, it had the disadvantage of long encryption and decryption time. In future research, we will apply the idea of block processing to optimize the proposed algorithm. Meanwhile, considering that the hyper-chaotic system has the same excellent chaotic characteristics as a fractional order chaotic system, we will try to design an image encryption algorithm based on a hyper-chaotic neural network. In recent years, machine learning and deep learning have performed well in the field of image processing. Thus, we will try to introduce these techniques to design a secure and efficient color image encryption algorithm.

## Figures and Tables

**Table 1:** DNA encoding rule.

Rule	1	2	3	4	5	6	7	8
A	00	00	11	11	01	01	10	10
G	01	10	01	10	11	00	11	00
C	10	01	10	01	00	11	00	11
T	11	11	00	00	10	10	01	01

**Table 2:** DNA XOR operation.

XOR	A	T	C	G
A	A	T	C	G
G	G	C	T	A
C	C	G	A	T
T	T	A	G	C

**Table 3:** The PSNR value between decrypted image and plain image.

Images	PSNR		
	R	G	B
Lena	Inf	Inf	Inf
Baboon	Inf	Inf	Inf
Pepper	Inf	Inf	Inf

**Table 4:** Chi-square for plain image and its corresponding cipher image.

Image	Plain Image			Cipher Image		
	R	G	B	R	G	B
Lena	5.93 × 10${}^{4}$	3.13 × 10${}^{4}$	8.09 × 10${}^{4}$	215.2266	224.8203	245.3828
Baboon	2.60 × 10${}^{4}$	4.28 × 10${}^{4}$	2.84 × 10${}^{4}$	282.2500	244.5000	278.4063
Pepper	5.08 × 10${}^{4}$	3.29 × 10${}^{4}$	8.68 × 10${}^{4}$	248.8594	207.5859	278.6061

**Table 5:** The coefficients of correlation between adjacent pixels of the plain image and its corresponding cipher image.

Image	Direction	Plain Image			Cipher Image		
		R	G	B	R	G	B
Lena	Horizontal	0.9706	0.9733	0.9452	0.0009	−0.0012	0.0007
	Vertical	0.9450	0.9450	0.8941	−0.0005	−0.0016	−0.0010
	Diagonal	0.9187	0.9228	0.8578	−0.0004	−0.0009	0.0001
Baboon	Horizontal	0.9407	0.9094	0.9496	0.0010	−0.0003	0.0022
	Vertical	0.9505	0.9194	0.9536	0.0023	0.0033	0.0023
	Diagonal	0.9060	0.8539	0.9145	0.0029	−0.0030	0.0001
Pepper	Horizontal	0.9242	0.9659	0.9275	0.0043	0.0019	0.0024
	Vertical	0.9290	0.9646	0.9269	−0.0006	−0.0009	−0.0001
	Diagonal	0.8766	0.9403	0.8783	−0.0013	0.0026	0.0017

**Table 6:** Comparison on coefficients of correlation for Lena image.

Algorithm	Direction	R	G	B
Proposed	Horizontal	0.0009	−0.0012	0.0007
	Vertical	−0.0005	−0.0016	−0.0010
	Diagonal	−0.0004	−0.0009	0.0001
Ref. [33]	Horizontal	0.0091	−0.0012	−0.0223
	Vertical	−0.0123	0.0047	−0.0057
	Diagonal	0.0258	0.0188	−0.0142
Ref. [34]	Horizontal	0.0014	0.0033	0.0021
	Vertical	0.0048	−0.0006	0.0002
	Diagonal	0.0002	0.0048	−0.0040
Ref. [35]	Horizontal	−0.0002	−0.0015	−0.0034
	Vertical	−0.0001	0.0041	−0.0056
	Diagonal	−0.0031	−0.0004	−0.0003
Ref. [36]	Horizontal	0.0083	−0.0054	−0.0010
	Vertical	−0.0049	0.0100	0.0124
	Diagonal	−0.0095	−0.0017	−0.0042
Ref. [37]	Horizontal	0.0021	0.0053	0.0011
	Vertical	0.0030	−0.0002	−0.0023
	Diagonal	0.0060	0.0034	−0.0005

**Table 7:** NPCR and UACI between the cipher image encrypted by *K* and the cipher image encrypted by $K_{1}$.

Image	NPCR (%)			UACI (%)		
	R	G	B	R	G	B
Lena	99.6277	99.6140	99.5636	33.4383	33.4221	33.4527
Baboon	99.6338	99.5865	99.5636	33.3224	33.6109	33.4995
Pepper	99.5636	99.6185	99.5972	33.5139	33.5660	33.5406

**Table 8:** NPCR and UACI between the image decrypted by the wrong key and the plain image.

Index	Type	Component	Image		
			Lena	Baboon	Pepper
NPCR (%)	Calculated value	R	99.6262	99.6155	99.6140
		G	99.5697	99.5804	99.6490
		B	99.6780	99.5621	99.5651
	Ideal value	R			
		G		99.6094	
		B			
UACI (%)	Calculated value	R	32.7627	29.5176	28.7423
		G	30.4562	27.8932	33.4938
		B	27.8725	30.4958	34.0249
	Ideal value	R	32.6967	29.4993	28.7532
		G	30.5401	27.8160	33.4662
		B	27.7562	30.4805	34.0153

**Table 9:** Information entropy for plain images and cipher images.

Image	Plain Image			Cipher Image		
	R	G	B	R	G	B
Lena	7.2920	7.5658	7.0531	7.9976	7.9975	7.9973
Baboon	7.6634	7.3871	7,6646	7.9969	7.9973	7.9969
Pepper	7.3920	7.6150	7.1738	7.9973	7.9977	7.9969

**Table 10:** Information entropy comparison of Lena’s cipher image.

Algorithm	R	G	B
Proposed	7.9976	7.9975	7.9973
Ref. [33]	7.9975	7.9972	7.9977
Ref. [34]	7.9917	7.9912	7.9918
Ref. [35]	7.9975	7.9972	7.9969
Ref. [36]	7.9972	7.9972	7.9975
Ref. [37]	7.9972	7.9976	7.9975

**Table 11:** Differential attack results of color images.

Image	NPCR (%)			UACI (%)		
	R	G	B	R	G	B
Lena	99.6002	99.6506	99.6201	33.4866	33.4885	33.4942
Baboon	99.6170	99.5972	99.5956	33.4185	33.3454	33.4454
Pepper	99.6597	99.6323	99.6582	33.6292	33.4612	33.5380

**Table 12:** Comparison of differential attack results of Lena color image.

Algorithm	NPCR (%)			UACI (%)		
	R	G	B	R	G	B
Proposed	99.6002	99.6506	99.6201	33.4866	33.4885	33.4942
Ref. [33]	99.5590	99.5895	99.6063	33.5696	33.4967	33.5644
Ref. [34]	99.6243	99.6185	99.6280	33.4224	33.4361	33.4603
Ref. [35]	99.6124	99.6140	99.6201	33.4235	33.4838	33.5983
Ref. [36]	99.6078	99.6678	99.6078	33.5644	33.4458	33.5055
Ref. [37]	-	-	-	-	-	-

**Table 13:** The security analysis results of all-white cipher image and all-black cipher image.

Image	Component	Correlation Coefficients			Chi-Square	Information Entropy
		Horizontal	Vertical	Diagonal		
all-white	R	0.0038	0.0013	0.0032	212.8594	7.9977
	G	−0.0021	0.0016	−0.0004	255.8438	7.9972
	B	0.0035	−0.0029	0.0015	258.0000	7.9972
all-black	R	0.0032	−0.0024	0.0017	274.5547	7.9970
	G	−0.0004	−0.0005	−0.0017	269.8046	7.9970
	B	0.0007	0.0025	−0.0027	269.1016	7.9970

## Data Availability

Data sharing not applicable.

## Abbreviations

The following abbreviations are used in this manuscript:

CNN	Chaotic neural network
DNA	Deoxyribonucleic acid
HNN	Hopfield neural network
QR	Qatari Rial
LE	Lyapunov exponent
SE	spectral entropy
A	Adenine
G	Guanine
C	Cytosine
T	Thymine
SHA-256	Secure hash algorithm-256
PSNR	Peak signal-to-noise ratio
NPCR	Number of pixels change rate
UACI	Unified average changing intensity
SPN	Salt & pepper noise
GN	Gaussian noise
